# Importance of Investigating Occult Gallbladder Injury in Hepatic Trauma: A Case Report

**DOI:** 10.7759/cureus.85588

**Published:** 2025-06-09

**Authors:** Matheus de Oliveira Santos, Larissa S Coimbra, Aline Sardow, Rafael B Pelosi, Sandro Scarpelini

**Affiliations:** 1 Division of Trauma and Acute Care Surgery, Hospital das Clínicas da Faculdade de Medicina de Ribeirão Preto - Universidade de São Paulo (FMRP-USP), Ribeirão Preto, BRA

**Keywords:** blunt abdominal trauma, cholecystectomy, gallbladder injury, hepatic trauma, intramural hematoma

## Abstract

Gallbladder injury secondary to blunt abdominal trauma is a rare condition and may be missed during the initial evaluation. We present the case of a 65-year-old male patient with a history of chronic alcohol and tobacco use who was admitted two weeks after a physical assault, with facial trauma, abdominal pain, and hemodynamic stability. Initial imaging revealed a grade III hepatic injury and mild thickening of the gallbladder wall. The patient was initially managed conservatively. However, within 48 hours, he developed persistent right upper quadrant pain, low-grade fever, leukocytosis, and a positive Murphy’s sign. Abdominal ultrasound showed thickening of the gallbladder wall and intraluminal hyperechoic content, suggestive of clots. Laparoscopic cholecystectomy confirmed gallbladder contusion with an intramural hematoma and intraluminal clots, while the liver injury showed no active bleeding. The patient recovered uneventfully and was discharged the following day. This case highlights the importance of maintaining clinical vigilance in patients with blunt abdominal trauma, particularly when symptoms persist despite stable imaging findings. Gallbladder contusions may not be easily detected on imaging, and a high index of suspicion is crucial for timely diagnosis and treatment. Surgical intervention remains the definitive treatment and can prevent complications such as bile peritonitis or sepsis. This report adds to the limited literature on occult gallbladder injuries and emphasizes the importance of close follow-up and early surgical intervention in similar cases.

## Introduction

Hepatic trauma is one of the most frequent abdominal injuries in polytrauma patients. In contrast, associated injuries of the gallbladder are rare, occurring in approximately 2% of blunt abdominal traumas and may go unrecognized during the initial assessment, leading to complications if not promptly identified and treated [1,2].

Traumatic gallbladder injuries often present with nonspecific clinical signs, making early diagnosis challenging. Symptoms may be initially subtle or masked by concomitant injuries [1]. Imaging modalities such as ultrasound and computed tomography (CT) are critical for evaluation but findings can be inconclusive in the early phase, especially when there is no significant hemoperitoneum or bile leak [3].

Clinical vigilance remains crucial in cases of blunt abdominal trauma where initial imaging does not fully explain persistent or evolving symptoms. Progressive right upper quadrant pain, fever, and leukocytosis should raise suspicion for occult gallbladder injury [2]. In such scenarios, early surgical intervention - typically through laparoscopic cholecystectomy - may be necessary to prevent complications such as bile peritonitis or secondary infection [4].

This case highlights the diagnostic challenges associated with occult gallbladder injuries following blunt abdominal trauma and emphasizes the importance of clinical vigilance and timely surgical intervention to prevent morbidity.

## Case presentation

A 65-year-old male patient with no known comorbidities, and a history of chronic alcohol and tobacco use, was admitted to the Emergency Department two weeks after being assaulted. He presented with facial trauma, abdominal pain, and hemodynamic stability, without clear signs of peritonitis on initial physical examination.

**Figure 1:**
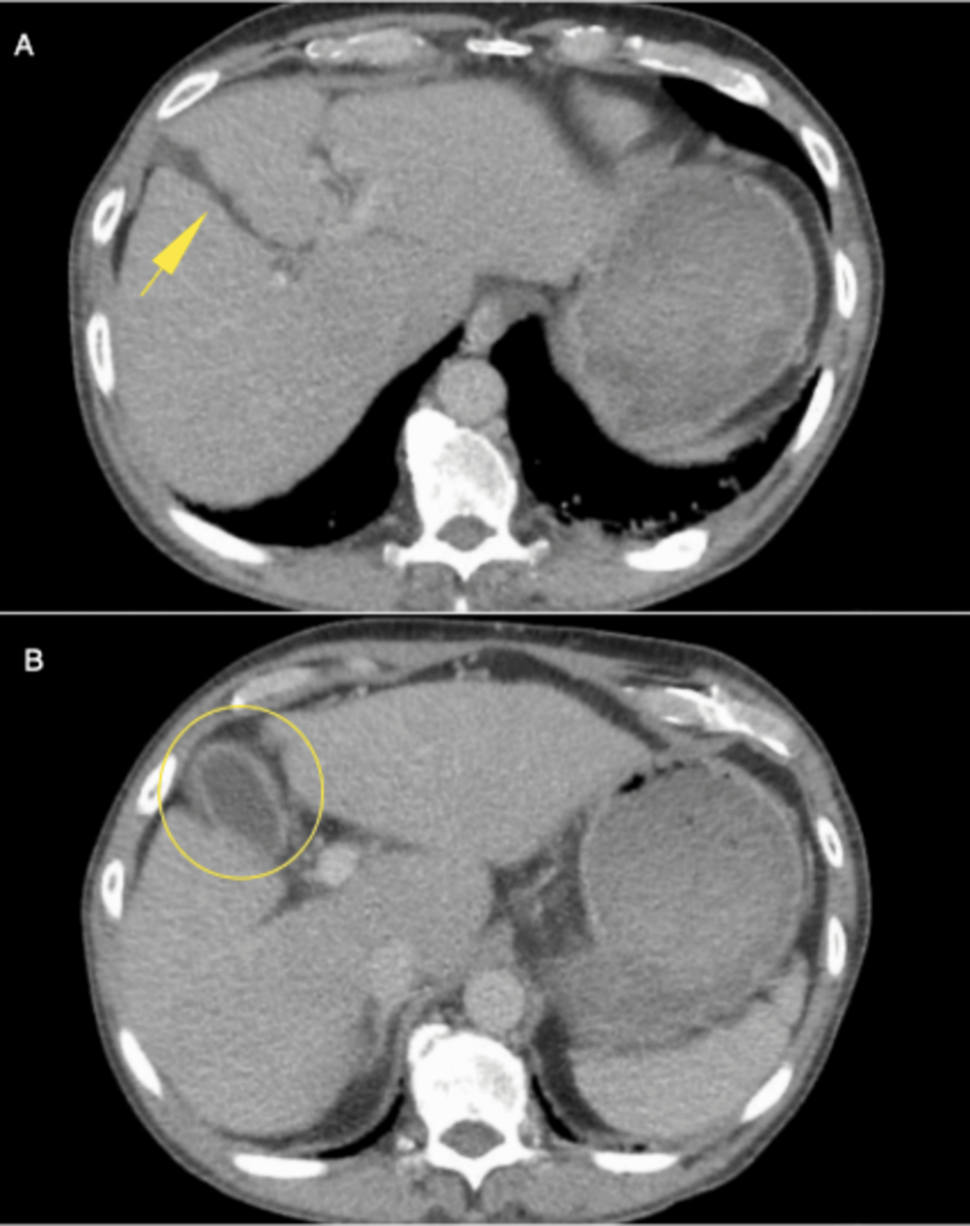
A) Contrast-enhanced CT showing grade III hepatic injury in segment V (yellow arrow); B) Contrast-enhanced CT showing mild gallbladder wall thickening (transparent yellow circle). CT, computed tomography

Contrast-enhanced CT revealed facial fractures, a small focus of subarachnoid hemorrhage, a grade III hepatic injury in segment V, and discrete thickening of the gallbladder wall (Figures 1A-1B). The patient was initially managed conservatively with clinical monitoring and analgesic support.

Within the first 48 hours, the patient developed persistent right upper quadrant pain, low-grade fever, and a positive Murphy’s sign. Laboratory tests revealed leukocytosis and elevated inflammatory markers. Abdominal ultrasound showed a normally shaped gallbladder with a thickened wall (6 mm) and hyperechoic images inside suggestive of clots or stones.

**Figure 2:**
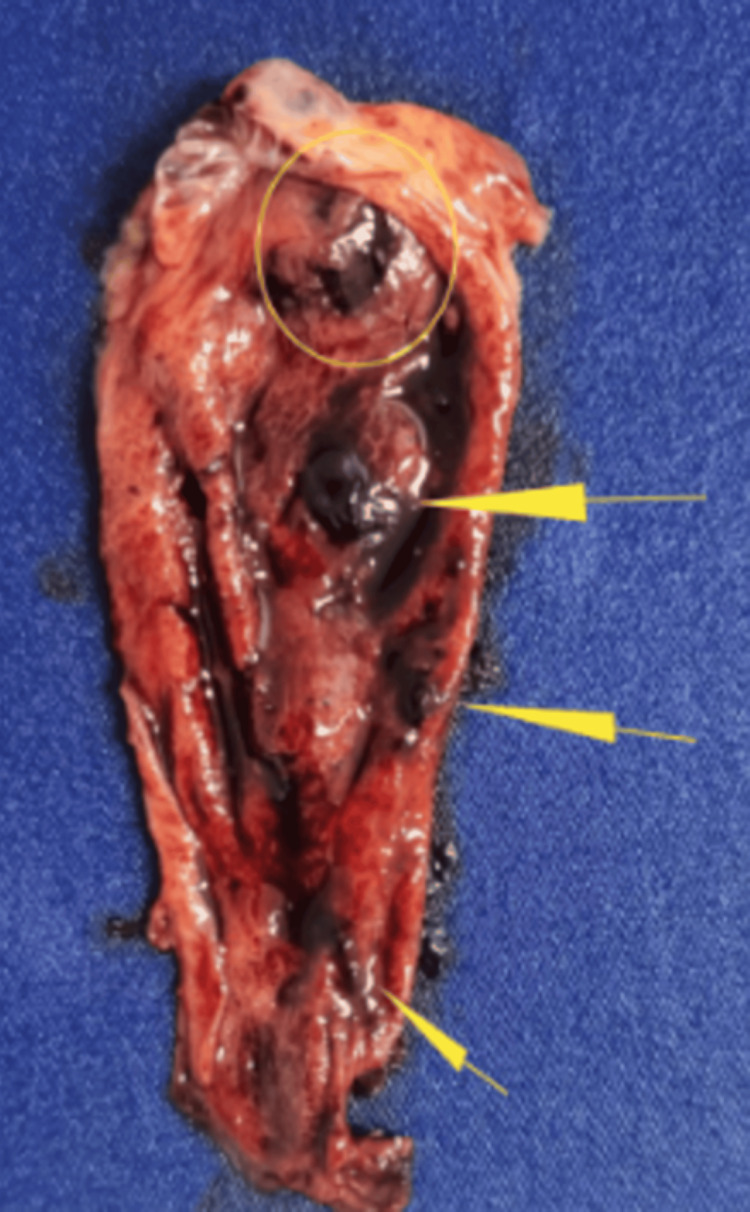
Surgical specimen image showing gallbladder with intramural hematoma (transparent yellow circle) and intraluminal clots (yellow arrows).

He underwent laparoscopic cholecystectomy, which revealed an intramural hematoma and a large amount of clots inside the gallbladder (Figure 2). The liver injury was organizing, with no active bleeding. The patient recovered well postoperatively and was discharged the following day.

## Discussion

Non-operative management is the gold standard for hemodynamically stable patients with liver trauma, strongly supported by the literature [5]. However, this case highlights the importance of continuous clinical surveillance, especially when symptoms persist.

Traumatic injuries of the gallbladder are rare due to the anatomical protection provided by the liver, ribs, and abdominal wall [6]. They represent less than 2% of blunt abdominal traumas [1], usually occurring in high-energy traumas or with associated injuries [7]. However, focal trauma directed at the right upper quadrant can compress the gallbladder against adjacent structures, leading to contusions, hemorrhage, or partial avulsion [8].

Injuries can be classified as contusion, laceration, or avulsion. This case fits the definition of a contusion with intramural hematoma and clots, which are often underdiagnosed in imaging studies [8]. Certain predisposing factors have been identified for gallbladder injury following blunt abdominal trauma: a thin-walled, normal gallbladder, a distended gallbladder, and alcohol ingestion [9].

Although CT and ultrasound are fundamental diagnostic tools, their sensitivity remains limited. Thus, in the setting of inconclusive imaging findings and clinical-radiological dissociation, surgical exploration becomes necessary to establish a definitive diagnosis [10]. The most common CT finding is pericholecystic fluid, although this is not specific for gallbladder injury and can be seen with other intra-abdominal injuries [11]. In our case, the persistence of pain, fever, and a positive Murphy’s sign indicated the need for intervention.

Surgical intervention allowed for a definitive diagnosis and treatment, preventing complications such as bile peritonitis or sepsis. Sharma reported that diagnosis was made intraoperatively in several cases of traumatic gallbladder injury [12], reinforcing the importance of clinical suspicion when symptoms persist.

This case adds to the scarce literature on occult gallbladder injuries after blunt trauma, highlighting the need for close clinical follow-up and a low threshold for surgical intervention.

## Conclusions

This case highlights the diagnostic pitfalls associated with blunt traumatic gallbladder injuries, a rare and often underrecognized entity. The persistence of clinical signs, despite often non-specific symptoms and imaging findings, should prompt high clinical suspicion and early surgical exploration to prevent adverse outcomes. Our patient's favorable evolution after laparoscopic cholecystectomy underscores the importance of not relying solely on imaging but integrating clinical evolution into decision-making. By reporting this case, we add to the scarce body of literature on occult gallbladder injuries, providing further evidence that delayed or missed diagnosis can be avoided through attentive follow-up and timely intervention. Although this is a single case report, with inherent limitations in generalizability, it highlights the need for further case series or retrospective studies to better characterize these injuries. This report assists clinicians in recognizing similar patterns in future cases, improving the diagnostic accuracy and management of this challenging clinical scenario.
